# Extracting information from the shape and spatial distribution of evoked potentials

**DOI:** 10.1016/j.jneumeth.2017.12.014

**Published:** 2018-02-15

**Authors:** Vítor Lopes-dos-Santos, Hernan G. Rey, Joaquin Navajas, Rodrigo Quian Quiroga

**Affiliations:** a Brain Institute, Federal University of Rio Grande do Norte, Natal, Rio Grande do Norte, Brazil; b Centre for Systems Neuroscience, University of Leicester, Leicester, UK; c Institute of Cognitive Neuroscience, University College London, London, WC1N 3AZ, UK

**Keywords:** Wavelet decomposition, Event-related potentials, EEG

## Abstract

**Background:**

Over 90 years after its first recording, scalp electroencephalography (EEG) remains one of the most widely used techniques in human neuroscience research, in particular for the study of event-related potentials (ERPs). However, because of its low signal-to-noise ratio, extracting useful information from these signals continues to be a hard-technical challenge. Many studies focus on simple properties of the ERPs such as peaks, latencies, and slopes of signal deflections.

**New method:**

To overcome these limitations, we developed the Wavelet-Information method which uses wavelet decomposition, information theory, and a quantification based on single-trial decoding performance to extract information from evoked responses.

**Results:**

Using simulations and real data from four experiments, we show that the proposed approach outperforms standard supervised analyses based on peak amplitude estimation. Moreover, the method can extract information using the raw data from all recorded channels using no a priori knowledge or pre-processing steps.

**Comparison with existing method(s):**

We show that traditional approaches often disregard important features of the signal such as the shape of EEG waveforms. Also, other approaches often require some form of a priori knowledge for feature selection and lead to problems of multiple comparisons.

**Conclusions:**

This approach offers a new and complementary framework to design experiments that go beyond the traditional analyses of ERPs. Potentially, it allows a wide usage beyond basic research; such as for clinical diagnosis, brain-machine interfaces, and neurofeedback applications requiring single-trial analyses.

## Highlights

• A decoding approach for extracting and quantifying information from ERPs is proposed.
• The proposed framework extracts more information than standard supervised approaches.
• The method allows analysis of multichannel signals.

## 1 Introduction

Event-related potentials (ERPs) are deflections in electrophysiological signals, such as electroencephalograms (EEGs), local field potentials (LFPs) or magnetoencephalograms (MEGs), which are triggered by external stimuli or internal cognitive processes (Freeman and Quian Quiroga, 2013; Regan, 1989). Due to the low signal-to-noise ratio of the ERPs, responses to several presentations are typically averaged to cancel out the background activity and improve the visualization of the evoked responses (Dawson, 1954). From the average ERPs, the standard approach is then to characterize the peak amplitude, latency, and topography of observed responses (Freeman and Quian Quiroga, 2013; Niedermeyer and Silva, 2005). Although this traditional analysis strategy has provided useful information about responses in different brain areas to various types of stimuli and tasks (Freeman and Quian Quiroga, 2013; Regan, 1989), it disregards information that may not be reflected by these quantifications based on the average responses (Quian Quiroga et al., 2007; Rey et al., 2015a, Rey et al., 2015b).

Previous attempts to extract information beyond the one provided by ensemble averaging have been, to a large extent, driven by the analysis of single trial evoked responses (Deweerd, 1981; Deweerd and Kap, 1981; Quian Quiroga et al., 2007; Walter, 1968). In this regard, the use of wavelet-based methods (Bartnik and Blinowska, 1992; Quian Quiroga et al., 2001; Thakor et al., 1993) has been particularly successful, mainly due to their ability to decompose signals into multiple scales, therefore being suitable for the analysis of ERPs, which typically contain waveforms of multiple frequencies (Quian Quiroga, 2000). We recently applied a wavelet denoising implementation (Ahmadi and Quian Quiroga, 2013; Quian Quiroga and Garcia, 2003) to extract the single-trial amplitudes and latencies of the N170 component (a negative deflection at ∼170 ms after the stimulus onset in the occipitotemporal cortex) recorded with scalp EEG while subjects observed pictures of faces and cars at the threshold of awareness (Navajas et al., 2013). Using this procedure, we were able to decode on a trial-by-trial basis the conscious recognition of the faces by the subjects, dissociating two different response patterns, one given by single trial amplitude differences and the other one given by differences in latency jitters, which we could not assess from the study of the average responses.

In spite of these advances, the analysis of single trial responses is still focused on quantifications based on the (single-trial) peak amplitude and latency of the ERPs, and does not typically consider: i) the specific morphology of the responses (i.e., the shape of the ERP); ii) information given by the combination of features from different evoked components; iii) information that may not be represented by peak responses (e.g., a DC shift) and iv) information that may be given by the combination of patterns at different recordings sites. For example, Jongsma et al. (2006) introduced a ‘learning-oddball’ paradigm and showed that the difference between two ERP components (the N2 and P3) was much more robust to distinguish two experimental conditions compared to each individual ERP. The problem is that finding such informative combinations is an *ad hoc* process that requires exhaustive searches and may be also prone to statistical biases, considering that an exhaustive search for informative combinations should be corrected for multiple comparisons. In addition, as discussed above, the study of single or multiple peak characteristics gives only a limited access to information that might be available to dissociate between different experimental conditions.

In recent years, multivariate pattern analysis (MVPA) techniques have been successfully applied in EEG and MEG (Cauchoix et al., 2014; Crouzet et al., 2015; King and Dehaene, 2014; Schönauer et al., 2017). These techniques look for patterns of neural activity considering all data available and define decision boundaries in a neural representational space that best distinguish different experimental conditions to be analyzed (Bray, 2009; Haxby et al., 2014). Thus, MVPA has the potential to capture the full spatiotemporal dynamic of signals like EEG (Parra et al., 2008). It is also more sensitive than multiple univariate comparisons, and it can be used for both, data driven exploratory analysis and hypothesis driven testing (Jamalabadi et al., 2016).

Nevertheless, the number of training data points is usually small with respect to the dimension of the neural representational space, and therefore, methods for feature selection are essential to avoid poor performance due to over-fitting the data with limited training samples. This has been performed based on *a priori* information, with the associated risk of biasing the resulting findings and even missing important information (Bray, 2009; Parra et al., 2008; Yang et al., 2012).

To overcome all these issues, we here propose a new MVPA method with an efficient dimensionality reduction step, allowing us to study the data recorded from all the electrodes without requiring *a priori* information. We call this the Wavelet-Information (WI) method. Based on an algorithm we recently proposed to extract information in time patterns of spike trains (Lopes-dos-Santos et al., 2015), the new method involves: i) decomposing individual responses with wavelets and using information theory (Shannon, 1948) to automatically identify a subset of coefficients carrying information about the stimuli or conditions (classes); ii) using these coefficients to train a classifier to predict classes and iii) quantifying information about the stimulus classes in the ERP responses based on the cross-validated performance of the classifier. This way, the method automatically extracts brain activity patterns that contrast different conditions/stimuli defined in the experimental design.

We validated the method using one simulated dataset and four different experimental datasets, and show that it gives significantly more information compared to the one provided by the study of single trial peak amplitudes, as used by Navajas et al. (2013). Moreover, we show that the method can be used to compute joint information from many channels in a completely unsupervised way, alleviating caveats and limitations that are inherent to the standard approach of a priori selecting regions of interests for the analysis. In fact, these selections tend to be hypothesis-driven and based on previous findings (thus limiting the possibility of new discoveries), and are typically mandatory in order to reduce the complexity of the computations and to avoid statistical issues due to multiple comparisons. Finally, we show that from the multichannel results it is possible to localize the times and electrodes providing informative patterns, and that the method does not show a deterioration of performance when increasing the number of channels, something that is common in classic information estimation approaches due to an increase of the dimensionality and complexity of the problem (Quian Quiroga and Panzeri, 2009).

## 2 Materials and methods

### 2.1 Simulated data

**Fig. 1:**
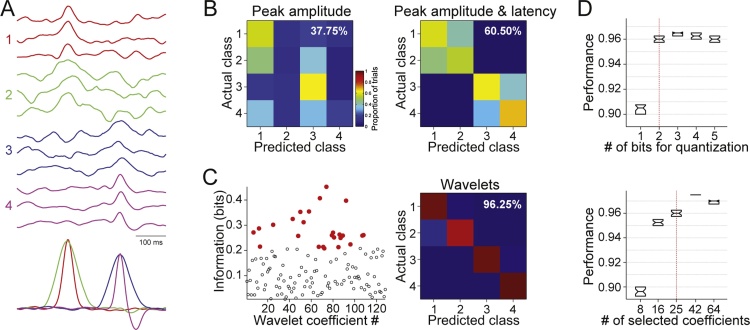
Peak analysis versus wavelet-based feature extraction. (**A**) Examples of single trials simulated for four hypothetical stimuli (classes), as labelled; along with average traces for each stimulus (100 trials each). (**B**) Confusion matrices for decoders trained with peak amplitudes (left) and peak amplitudes and latencies (right) when classifying the 100 trials per stimulus generated in **A**. Pseudocolors denote proportion of trials from a given class (rows) assigned to a given class (columns) by the decoder. Decoding performance of each classifier is displayed in the top right corner of each confusion matrix. (**C**) Left panel shows the estimated information of each wavelet coefficient. The best 25 coefficients (most informative) are displayed in red. Right panel displays the confusion matrix for the WI method using the same dataset as in **B**. (**D**) Performance with the simulated dataset for different values of the number of bits used for quantization (top panel) and the number of wavelet coefficients selected (bottom panel). Default values are indicated by red dashed lines. (For interpretation of the references to colour in this figure legend, the reader is referred to the web version of this article.)

We used simulated evoked potentials in order to illustrate the advantages of wavelet decomposition with respect to peak analyses (Fig. 1). We created a response pattern for each of four hypothetical stimuli. The waveforms of Stimulus 1, 2 and 3 consisted of a Gaussian waveform with a particular amplitude, latency and standard deviation (which relates to its frequency components). The pattern in Stimulus 4 was generated by a combination of two Gaussians. Considering a sampling rate of 256 Hz for the simulations, responses were 500-ms long. For each stimulus, 100 single trials were simulated. For each single trial, the Gaussian components were generated with a random jitter (±5 ms, uniformly distributed) and added to a background activity generated by EEG surrogates constructed from a real resting-state EEG recording (i.e. surrogate realizations keeping the amplitude and frequency distribution of a real EEG recording, including typical components such as alpha rhythms), as in previous works (Ahmadi and Quian Quiroga, 2013; Quian Quiroga and Garcia, 2003). The mean signal to noise ratio in each trial was set to 0.33 (power of the background noise was 3 times larger than the power of the patterns). Fig. 1A displays three examples of single trials for each stimulus (top), along with the average responses across 100 simulated trials (bottom).

### 2.2 Experimental data

We used 4 datasets to test the proposed method. Three of them consist of EEG recordings: Dataset **1** and **2** come from Visual and Auditory Oddball paradigms, respectively, previously reported in (Ahmadi and Quian Quiroga, 2013); and Dataset **3** comes from a face perception experiment, presented in (Navajas et al., 2013). Dataset **4** consists of local field potentials (LFPs) recorded from microelectrodes implanted in the medial temporal lobe (MTL) of humans during conscious recognition of visual stimuli (Quian Quiroga et al., 2008; Rey et al., 2014).

#### 2.2.1 Dataset 1: visual oddball

ERPs were recorded following a reversal of colors of a checkerboard pattern. Two stimuli were used: the Non-Target stimulus was presented in 80% of the trials (pseudorandomly selected) and consisted simply of a color reversal of checks; and the Target stimulus (the oddball) was presented in the remaining 20% of the trials and consisted of color reversals plus a half-check diagonal displacement. The presentation of pattern reversals was 1-s long and the inter stimulus interval varied pseudorandomly between 2 and 2.2 s. Subjects were instructed to fixate on a small red circle in the center of the screen and indicate the presence of the target stimulus by pressing a key. In total, 10 subjects responded to 250 trials in 14 sessions.

The EEG data was continuously recorded using 64 electrodes placed according to the 10/10 system (also known as MCN system, which stands for modified combinatorial nomenclature), band pass filtered between 0.1 Hz and 100 Hz and sampled at 256 Hz. The average across all channels was used as reference. In addition, trials with eye movements, blinks, and other artifacts were rejected offline by visual inspection.

#### 2.2.2 Dataset 2: auditory oddball

In this experiment, the Non-Target (also presented in 80% of the trials) and Target stimuli consisted of 2000-Hz and 1000-Hz tones, respectively. Nine subjects were instructed to press a key whenever they heard any of the stimuli (n = 9 sessions). Each stimulus was presented for 100 ms and the inter stimulus interval varied pseudorandomly between 1.5 and 1.7 s. As in the *Visual Oddball* experiment, subjects were instructed to fixate at a small red circle in the center of the screen. EEG recording acquisition and pre-processing was similar to the one described for Dataset 1.

#### 2.2.3 Dataset 3: face perception experiment

In this paradigm, trials comprised four steps: i) a fixation cross was presented for 500–700 ms; ii) this was followed by a brief flash of a face or a car presented for 57 ms; iii) then, a mask created with randomly shuffled pieces of different images was presented for 443 ms and iv) subjects reported whether or not they perceived a face using two buttons of a mouse (“seen” or “unseen” trials). In order to manipulate the visibility of the stimuli, we added zero-mean Gaussian noise with different variance levels. A single session was recorded for each subject (n = 22). The noise level was adjusted through a double-staircase procedure (Cornsweet, 1962) that kept running throughout the experiment. Upon completion of the experiment, we took all 250 trials where a face was presented and selected one level of noise that led to 50/50 recognition performance, in order to ensure that the comparison across “seen” and “unseen” trials was performed at constant retinal stimulation. EEG responses were recorded using the same equipment and same electrode set-up as in Datasets 1 and 2. Sampling rate was set at 256 Hz and signals were referenced to the average and filtered between 0.1 Hz and 70 Hz. In addition, trials with eye movements, blinks, and other artifacts were rejected offline by visual inspection.

#### 2.2.4 Dataset 4: human LFPs

Intracranial recordings were obtained in 12 sessions from 5 patients with pharmacologically intractable epilepsy. Depth electrodes were surgically implanted to determine seizure focus for possible resection (Rey et al., 2015a, Rey et al., 2015b) and their location was exclusively determined by clinical criteria. Each electrode bundle had a total of 8 active recording microwires and a local reference. Electrodes were placed mainly in the medial temporal lobe (MTL), with 8 bundles placed at the hippocampus, 5 at amygdala and 5 at the entorhinal cortex. One patient was implanted with a total of 7 probes, whereas all remaining patients had 8 probes in total. Target areas outside the MTL included the temporal gyrus, the cingulate cortex, the supplementary motor area, the orbitofrontal cortex, and the temporal pole.

Trials involved the presentation of pictures in pseudorandom order followed by a mask, consisting of randomly shuffled pieces from different images (Quian Quiroga et al., 2008). Each of 16 pictures was presented 8 times for each of four different durations: 33 ms, 67 ms, 100 ms and 250 ms. The total duration of the trials (picture + mask) was 500 ms. At the end of the trials, subjects indicated if they recognized the picture by pressing left or right arrow keys. Trials were classified as ‘recognized’ or ‘non-recognized’ according to these reports, with a percentage of recognized trials across sessions of 83.1 ± 3.6. The LFPs were constructed by filtering the raw data between 2 and 120 Hz and then reducing the sampling rate to 256 Hz. Epochs of 1 s after stimulus onset were extracted for each trial.

In this dataset, it was possible to identify single cell responses to at least one picture in 37 different channels (Rey et al., 2014). LFPs recorded from electrodes with at least one responsive cell were used in the analysis shown in Fig. 3, Fig. 4, and in the single channel set in Fig. 5.

### 2.3 Description of the WI method

The proposed method has three main steps: **i**) *Wavelet decomposition*, which is a time frequency decomposition of the single trial EEG traces; **ii**) *Wavelet coefficient selection*, which aims at identifying which wavelet coefficients carry meaningful information and **iii**) *Decoding*, which classifies trials based on the selected coefficients. Thus, in general terms, the method has a *feature extraction* stage (comprised by steps **i** and **ii**) and a *classification* stage (step **iii**). These steps are discussed below in more detail.

#### 2.3.1 Wavelet decomposition

Given a signal x(t) and a wavelet function ψ*_a,b_*, the continuous wavelet transform (CWT) can be defined as:

$X\left(a,b\right)=x,\psi_{a,b}$, with

ψa,b=|a|−12ψ(t−ba),

where <,> denotes the inner product and *a* and *b* are the scale and translation parameters, respectively. The scale parameter dilates or compresses the wavelet function, and thus, it defines which time scale of the signal is captured by the coefficient: dilated wavelet functions capture slow frequency components, whereas compressed versions capture high frequency patterns. The translation parameter shifts the wavelet function in time. In sum, wavelet coefficients characterize features in the signal of interest by decomposing its activity into patterns at different times and frequency ranges.

In order to avoid redundancy, without loss of information it is possible to define the wavelet transform at ‘dyadic’ set of scales and times, defined as a_j_ = 2^j^ and b_j,k_ = 2^j^k, respectively, where *j* is an integer that defines the scale and *k* is an integer that defines time location (Strang and Nguyen, 1996). This dyadic wavelet transform can be implemented in a recursively and fast algorithm, named *multiresolution decomposition*, which decomposes the signal into a set of detail scales and a final approximation (Strang and Nguyen, 1996).

Here, we followed the same implementation as in Lopes-dos-Santos et al. (2015) using a five-scale multiresolution decomposition with Haar wavelets, with an approximate frequency support of: 64–128 Hz (D1), 32–64 Hz (D2), 16–32 Hz (D3), 8–16 Hz (D4), 4–8 Hz (D5), and 0–4 Hz (A5). The application of more complex wavelet functions (such as quadratic B-splines) failed to improve the performance (not shown), therefore we kept the Haar mother wavelet in the final implementation for its simplicity. At first glance, this result seems counter-intuitive due to the square nature of the Haar function as opposed to the smooth and sinusoidal shape of other mother wavelets that would be more similar to the waveform of the ERPs. However, note that, rather than providing a faithful reconstruction of the signal (as in denoising methods), the purpose of the decomposition here is to identify features that can show maximal contrast between different stimuli or conditions. In fact, Haar wavelets have been shown to be particularly efficient for pattern recognition applications, such as classification of spike trains and spike sorting (see Discussion).

#### 2.3.2 Selection of wavelet coefficients

The multiresolution decomposition does not change the dimensionality of the original signal, i.e., from N data points we obtain N wavelet coefficients. In this step, we aim to perform an efficient dimensionality reduction by automatically identifying and selecting the coefficients that carry information about the stimulus/condition to be decoded. To this end, we computed the mutual information between each individual coefficient and the stimulus/condition, defined as (Shannon, 1948):

$$I_{S,w_{a,b}}=\underset{S,w_{a,b}}{\sum }P\left(S,w_{a,b}\right)log_{2}\frac{P\left(S,w_{a,b}\right)}{P\left(S\right)P\left(w_{a,b}\right)},$$

where S is the set of stimuli/conditions, *w*_a,b_ is the set of values of the corresponding wavelet coefficient, P(S) and P(*w*_a,b_) are the probabilities of having stimulus S and coefficient *w*_a,b_, respectively, and P(S,*w*_a,b_) is the corresponding joint probability. For each wavelet coefficient *w*_a,b_, the probability of the response P(*w*_a,b_) was estimated by dividing the values of *w*_a,b_ into 4 equally spaced bins, i.e. using 2 bits for quantization. Note that, at this point, we do not aim at accurately estimating the information of each individual coefficient; we simply need to rank them and feed the most informative ones to the decoder algorithm.

Since single trial recordings are typically noisy, we estimated the information carried by each component based on sub-ensemble average realizations. Specifically, we computed the mutual information based on 200 sub-ensemble averages from each condition, each of them constructed by averaging 30 randomly selected trials (with replacement) from the respective conditions. The 25 coefficients with the largest estimated information were further used for decoding (see Fig. 1C). Importantly, the whole selection of coefficients is strictly performed without taking into account the trial that will be decoded by the classifier in order to avoid upward bias in the classification performance (see below).

#### 2.3.3 Decoding

We used a uniform-prior naive Bayesian decoder in order to assign single trials to different conditions. The inputs to the classifier were the selected wavelet coefficients, as described in the previous step. To avoid overfitting, we used a leave-one-out cross-validation procedure: we classified each trial at a time, using the remaining trials to compute the sub-averages for information estimation and coefficient selection. Therefore, when classifying a given trial, all the information used to train the classifier comes from other trials. Decoding performance was defined as the proportion of trials correctly classified.

In Dataset 1 and 2, we trained classifiers to distinguish between the presentation of Target and Non-Target stimuli, using 1-s windows after stimulus onset of the EEG signals. This task typically elicits a P300 response, so we used the occipital electrode O1 and central electrode Cz for Datasets 1 and 2, respectively (unless stated otherwise), as in previous works (Ahmadi and Quian Quiroga, 2013). In Dataset 3, we trained classifiers to predict the conscious perception of faces by the subjects based on 500-ms EEG responses after stimulus onset. We used electrode PO8, unless stated otherwise, since this channel elicited stronger N170 responses in this experiment (Navajas et al., 2013).

#### 2.3.4 Single trial peak detection

We compared the information provided by the WI method to the one obtained with an analogous implementation but using the single trial peak amplitudes for decoding. For obtaining the single trial peak amplitudes we used a previously proposed wavelet denoising implementation that has been shown to improve the extraction of single trial components of ERPs (Quian Quiroga and Garcia, 2003). This method identifies coefficients related to the ERPs, by comparing the post-stimulus wavelet coefficients with the distribution of baseline values, and then reconstructs the single trial traces from these coefficients (Ahmadi and Quian Quiroga, 2013).

For the oddball data, before computing ensemble averages, the individual trials were detrended as this improved further the single trial peak estimation. No detrending was used with the WI method. To classify trials as Target and Non Target we used the single trial peak amplitude of the P3 response, which is the component that clearly separates both conditions in the ERP averages (Polich, 2007). The single trial P3 amplitude was defined as the local maximum between 350 and 700 ms (visual oddball) and 200 and 500 ms (auditory oddball) after stimulus onset.

For the face perception experiment we used the amplitude of the denoised single trial N170 component, defined as the local minimum between 120 and 200-ms after the stimulus onsets, as in (Navajas et al., 2013), to decode whether the subject recognized a face or not. Due to the use of a local reference for the LFP data, the polarity of the ERP varied (we observed a positive peak in 65% of the cases and a negative peak in the remaining 35%) and we therefore defined peak amplitude as the maximum/minimum between 150 and 350 ms after stimulus onset, for the positive/negative average ERP responses.

#### 2.3.5 Multichannel WI implementation

In order to use more than one channel for decoding, we: i) estimated the information of the wavelet coefficients from each channel individually, ii) ranked the coefficients from all channels in terms of their information, and iii) selected the 25 most informative coefficients from this pool. Note that, we did not force the decoder to use coefficients from every channel, instead, we always selected the best coefficients regardless of their spatial distribution. For each dataset, we compared results using all channels with the ones obtained from the *a priori* most informative channel, which was used to evaluate single channel performance in Fig. 3, Fig. 4. We also compared results with an *a priori* selection of 8 informative channels (based on the literature and proximity to the selected single channel): the midline electrodes (Oz, Cz, Pz, Fz, FCz, CPz, POz and AFz) for the oddball datasets; the occipitotemporal channels (PO8, P8, PO7, P7, O1, TP7, O2 and TP8) for the face perception dataset and the set of responses in the 8 microwires coming from the same bundle for the LFP dataset.

For the Dataset 4, channel aggregation was used for the statistical analysis in Fig. 5. The 37 single channels analyzed, came from 24 bundles in 12 different sessions. Then, in order to perform the paired sign tests between the different sets of channels (1 channel, 8 channels, all channels), the median performance (per bundle/session) was computed on the set with more elements. For example, the 37 performances obtained in the single channel case were converted into 12 (to compare with the all channels case for each session) by computing the median performance across all the single channels within each session.

#### 2.3.6 Assessment of statistical significance

To assess the statistical significance of the decoding performances, we rerun the method 100 times after randomly shuffling the trial classes. Thus, we use performances obtained from the “shuffled” data (surrogates) to construct a null hypothesis distribution of performance. Hence, we regard the proportion of shuffled performances above the original performance as its p-value (i.e., the probability that the observed performance was obtained by chance).

## 3 Results

### 3.1 Illustration of the method

Standard techniques for the study of ERPs focus on their amplitude and latency, missing information in the waveform of the responses. This issue is illustrated in Fig. 1 with simulated datasets (see Materials and Methods). The simulated responses to Stimulus 1 and 2 (red and green, respectively) have similar peak latencies and amplitudes; whereas the ones to Stimulus 3 and 4 (blue and magenta, respectively) present slightly smaller amplitudes and longer latencies. Thus, by design, peak information (latency and amplitude) can only distinguish Stimulus 1 and 2 from 3 and 4. Decoding results can be visualized as confusion matrices, where each entry denotes the probability of a trial from a given class *i* (rows) being classified as class *j* (columns). Thus, perfect decoding leads to a matrix with ones in the main diagonal. Fig. 1B shows the confusion matrices obtained from the decoder, trained with peak amplitudes (left) or with peak amplitudes and latencies (right). Notice that peak amplitude decoding shows a performance above chance (37.7% vs. 25%), but with many trials being misclassified. This shows that, although peaks of Stimulus 1 and 2 are higher than the ones from Stimulus 3 and 4 (on average), this information could not be retrieved on a single trial basis. The decoding performance increases (up to 60.5%) when both the single trial amplitude and latency are considered (Fig. 1B, right), given that the decoder can now distinguish between the early (stimulus 1 and 2) and the late peaks (Stimulus 3 and 4). However, the decoder still could not distinguish between the stimuli in each of these two subsets. This is consistent with the average responses (bottom of Fig. 1A), where it is clear that (by construction) Stimulus 1 and 2 and Stimulus 3 and 4 can only be distinguished based on the shape of the responses. In the example presented here, the WI method leads to a much higher decoding performance (96.2%, right panel in Fig. 1C). This is because wavelet coefficients captured information not only about the peak amplitudes and latencies, but also about the shape of the ERPs (Fig. 1C left).

Finally, we used the simulated dataset to evaluate the robustness of the method with respect to the parameters used for the selection of wavelet coefficient: number of bits for quantization when estimating information of individual wavelet coefficients, number of sub-ensemble averages and trials per average (see 2.3.2 section for more details), and number of selected wavelet coefficients. When evaluating the performance for a certain parameter, the remaining ones were fixed to their default value: 2 bits for quantization, 200 sub-ensemble averages of 30 trials each, and 25 wavelet coefficients. We found that the performance was only affected by less than 2% within a wide range of values for the number of sub-ensemble averages (50–400) and trials per sub-average (10–50). Furthermore, increasing quantization resolution by more than 2 bits (i.e. dividing values in more than 4 bins) did not increase performance (Fig. 1D, top). Notice that the purpose of this estimation is to identify informative coefficients rather than accurately estimating the mutual information between the coefficients and stimuli/conditions. Finally, we evaluated the number of wavelet coefficients selected (Fig. 1D, bottom). We found that performance decreased when a small number of coefficients was used, but by using at least 1/8 of the total number of coefficients led to a good and stable performance. Overall, the method is robust with respect to the choice of parameters as long as extreme values for the parameters are avoided.

### 3.2 Performance with real data

**Fig. 2:**
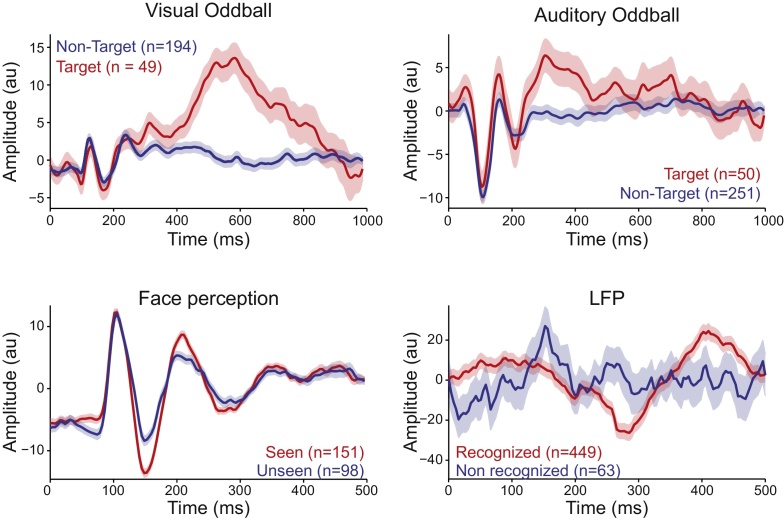
Description of datasets. Representative examples from each dataset used, as labelled. ERPs (mean ± SEM) from stimuli/conditions to be decoded are shown. Number of trials averaged on each trace are displayed in brackets.

**Fig. 3:**
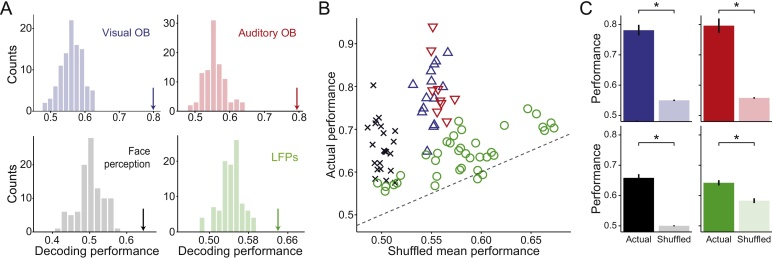
Validation of the method on single channels. (**A**) Each panel shows the decoding performance (indicated by arrows) of a representative session from each dataset along with performances obtained after shuffling classes-trials relations (histograms). (**B**) Decoding performance of each session in all datasets (vertical axis) paired with their corresponding shuffling mean performance (horizontal axis). Dashed line represents y = x, i.e., actual performance equal to shuffling mean performance. Datasets are displayed in different colors, as labelled. (**C**) Each panel shows the actual performances and mean shuffling performances (mean ± SEM) of a dataset. Color codes used are the same as in **A** and **B**. Actual performances are significantly higher than mean shuffled performances in all datasets (paired sign test; p = 3.9 × 10^−3^, p ∼ 10^−4^, p ∼ 10^−7^, p ∼ 10^−8^, for the auditory and visual oddball, face perception and LFPs datasets, respectively).

The example shown in Fig. 1 is based on synthetic data. To evaluate the performance of our algorithm on real data, we applied the WI method to three EEG datasets (Visual Oddball, Auditory Oddball, and Face Perception) and one LFP dataset (see Methods for details about the experimental designs). Each dataset consists in two experimental conditions, and the objective is to decode them with the highest possible performance. In Fig. 2, we plot the ERPs elicited by the two experimental conditions that will be compared. Data in Fig. 2 comes from representative participants: on each dataset, we selected the sessions that led to median performance using the WI method. Fig. 3A shows the decoding performance for these selected sessions. Actual performances are indicated by arrows and the histograms show the null distribution of decoding performances obtained from surrogated data (see Methods). Note that, for these examples, decoding performances were above the null distribution in all datasets (p < .01). In fact, the WI method showed significant decoding performance in the vast majority of sessions in all datasets. In both the Auditory (n = 9 sessions) and Visual Oddball (n = 14 sessions) datasets, this was the case for all sessions (p < .01 for all cases). For the Face Perception dataset, the decoding performance of our method was above the entire null distribution (p < .01) in 14 out of 22 sessions (63.64%), and in one participant, the observed decoding performance was close to significance (p = .06). With the LFP dataset, 19 out of 37 cases (51.35%) presented a performance above the entire null distribution (p < .01), and 22 cases (59.49%) exhibited a performance above the 95th percentile of the distribution (p < .05).

**Fig. 4:**
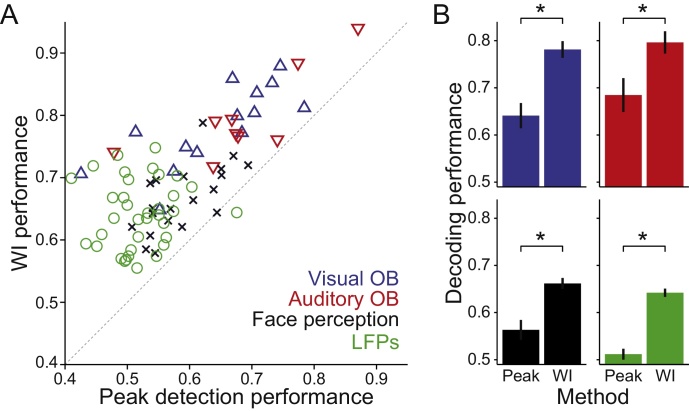
Comparison with the semi-supervised method. (**A**) Decoding performances obtained with peak detection after wavelet denoising and detrending (horizontal axis) and with the proposed method (vertical axis) for all sessions from all datasets, as labelled. Dashed line indicates x = y. (**B**) Each panel displays performances of both methods (mean ± SEM) in a dataset. Color code is same as in **A**. WI performance was always significantly larger (paired sign tests; p ∼ 10^−4^, p = 1.95 × 10^−3^, p ∼ 10^−7^, p ∼ 10^−10^, for the visual and auditory oddball, face perception and LFPs datasets, respectively).

**Fig. 5:**
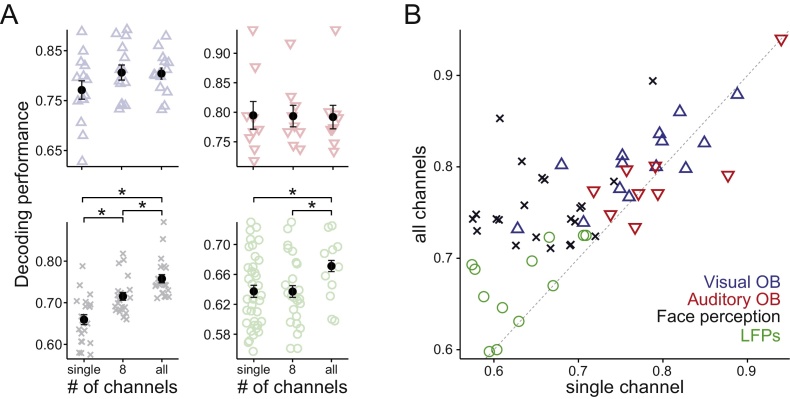
Performance of decoding with simultaneous channels. (**A**) Each panel shows decoding performance obtained when one, eight or all channels are used. Light marks show individual performances. Dark filled circles denote average performance and bars indicate SEM. No comparison was significant in both visual and auditory oddball datasets (p > .05). All comparisons in the face perception experiment were significant (paired sign tests, p ∼ 10^−4^, p ∼ 10^−4^, p ∼ 10^−4^, for single vs 8, single vs all, 8 vs all channels, respectively). For the LFP dataset, only single vs 8 and 8 vs all channels were significantly different (paired sign tests, p = 0.012 and p = 6.3 × 10^−3^, respectively; see text for details on how LFP performances were paired). Color codes are same as in **B**. (**B**) Performance of all sessions/responses from all datasets when a single channel (horizontal axis) or all channels (vertical axis) were used.

Fig. 3B shows the decoding performances from all sessions and datasets (vertical axis) against the mean performance of their corresponding null distributions (horizontal axis). Note that virtually for every session, the WI performance was above the one expected by chance (i.e. nearly all entries are above the diagonal, marked with a dashed line). Fig. 3C shows the average decoding performances, which for all datasets were significantly larger than the ones of the shuffled surrogates (paired sign test; p = 3.9 × 10^−3^, p ∼ 10^−4^, p ∼ 10^−7^, p ∼ 10^−8^, for the Auditory and Visual Oddball, Face Perception and LFPs datasets, respectively).

When applied to surrogated data, the WI method often led to a performance above 50%. This can be observed by looking at the horizontal axis in Fig. 3B, where most data points are above 50%. In principle, this could imply that our method has an intrinsic upward bias of classification. Alternatively, it could simply reflect that our datasets have an unbalanced number of trials on each class. In fact, the only dataset that has approximately equal number of trials per class supports this second alternative (Face perception dataset, black crosses in Fig. 3B). To confirm that our method does not have an intrinsic upward bias of classification, we subsampled the class with largest number of trials in unbalanced examples and recomputed the null distribution using surrogated data. Using a balanced number of trials per class, we found that our method led to a surrogate performance close to 50%.

### 3.3 Comparison with single trial peak amplitude decoding

So far, we have shown that the WI method leads to above-chance decoding performance in both synthetic and real data. Using simulations, we also observed that the WI method outperformed the standard approach of analyzing peak amplitudes and latencies (Fig. 1B, C). However, this was expected, as the shape of the synthetic ERPs provided additional information by construction (Fig. 1A). Here, we ask if this is also the case in real data. We compared decoding performances obtained with the WI method to the ones obtained using single trial peak amplitude estimation. We have not used the single-trial latency data as, in our 4 datasets, latencies did not improve decoding performance when compared to using only peak amplitudes. The left panel of Fig. 4 displays performances obtained by the WI method against the ones obtained with the peak detection method (see Methods) for all sessions in the 4 datasets. The WI method provides a higher decoding performance in virtually all cases (nearly all entries above the diagonal). Panels on the right display the mean performances for each dataset and method. For all four datasets, the WI method significantly outperformed the peak detection method (paired sign test; p ∼ 10^−4^ for the Visual Oddball, p = 3.9 × 10^−3^ for the Auditory Oddball, p ∼ 10^−7^ for the Face Perception dataset, and p ∼ 10^−10^ for the LFPs). This is despite the WI method attained these results in a fully unsupervised way, whereas the peak amplitudes estimation always involved at least one supervised step. For example, the experimenter needs to know a priori on each dataset the spatio-temporal distribution of the relevant ERPs (e.g. right occipito-temporal cortex in the 150–200 ms for the Face Perception dataset). Furthermore, to efficiently extract the single-trial peak amplitudes, signals often require pre-processing steps such as detrending the data. In fact, we observed that skipping this step led to a decrease in decoding performance of between 5 and 10% in the two Oddball datasets. No such preprocessing steps were necessary when using the WI method.

### 3.4 Combining information from multiple channels

Next, we studied if the WI method could be applied to data coming from multiple channels. For this, we calculated the information in the wavelet coefficients for each channel separately and ranked all wavelet coefficients together. Then, we took a selection of the most informative coefficients from all channels. The rationale of this procedure was to evaluate whether information from channels other than the one selected a priori could improve decoding performance. Fig. 5A shows the performance of the method for each dataset when using one channel (as before), when using a selection of 8 channels (see Methods), and when all channels were used. Fig. 5B shows, for all sessions and datasets, the single-channel decoding performance against the performance obtained using all channels.

In principle, adding more channels could lead to a decrease in decoding performance due to an increase in the complexity of the data, a well-studied phenomenon known as the “curse of dimensionality”. However, increasing the number of data points by 64-fold did not lead to a decay in performance of the WI method. On the contrary, we observed that, with increasing number of channels, performance remained flat in two datasets (top panels in Fig. 5A), and in the two other datasets performance increased (bottom panels in Fig. 5A). This is because our method performs an efficient dimensionality reduction by selecting only the most informative wavelet coefficients before the decoding step.

In the two Oddball paradigms, the presentation of an infrequent stimulus (i.e., the target) triggers a slow positive deflection in the EEG signal at >300 ms. This is a large global potential, that can be well-represented with a single central electrode (see top panels in Fig. 2). In fact, because this response is presumably the only neural process that distinguishes target from non-target sensory stimuli, adding more channels can only provide information that is redundant with the one obtained from a central channel. Consistent with these observations, we found no significant changes in performance when considering more channels both in the Visual and Auditory Oddball paradigms.

In the Face Perception dataset, we observed a significant increase in performance with increasing number of channels (paired sign tests, single vs. 8 channels p ∼ 10^−4^, single vs. all channels p ∼ 10^−7^, and 8 vs. all channels p ∼ 10^−4^). Previous studies have argued that an occipito-temporal EEG component, the N170, triggers face perception (Rossion, 2014). Although this component is stronger in the right hemisphere, it can also be detected in contralateral channels (Rossion et al., 2003). We believe that face-selective neural sources from the left hemisphere could have contributed to the increase in decoding performance observed between the single-channel and 8-channel sets. Moreover, slight cap misplacements and subject-to-subject variability might lead to individual differences in the location of the N170 peak activations, which can only be captured when taking a larger selection of channels. However, one intriguing aspect of our data is that we observed a substantial increase in performance, compared to the 8-channel set, when we considered all channels together. In principle, this could be attributed to face-selective neural processes outside the occipito-temporal cortex. Alternatively, it could reflect that other occipito-temporal electrodes that were not selected in the 8-channel set provided all the remaining information. To disambiguate these possibilities, we considered a larger set in which we included the 8-channel selection used before and 3 other occipito-temporal sites (P9, P10, and Iz, leading to a total of 11 channels). Using these 11 channels led to a significant increase in performance compared to the original 8-channel set (paired sign test, p ∼ 10^−4^), even to match the one obtained with the 64-channel set (p = 0.19). Thus, occipito-temporal electrodes alone could achieve the same performance as the whole electrode set. This finding provides further support to the view that face perception is triggered by activity in the occipito-temporal cortex.

The LFP dataset is based on intracranial recordings with depth electrodes mostly located in the MTL. However, several electrodes were placed in areas such as the temporal gyrus, the orbitofrontal cortex, the cingulate cortex, among others (see Methods for details). We found that using 8 channels did not change performance compared to using a single channel, but using all channels led to a significant increase (paired sign tests, single vs. 8 channels p = 1, single vs. all channels p = 0.0117, 8 vs. all channels p = 0.0063). Due to the nature of our recordings, the single and 8-channel datasets were all located in the same area of the MTL. Therefore, all electrodes in the 8-channel set might have provided redundant information to classify the two experimental conditions. This is indeed consistent with our previous findings showing global LFP deflections in the MTL for recognized stimuli (Rey et al., 2014). When using the entire dataset, information from other brain regions became available to our classifier, and this might explain why we observed an increase in decoding performance.

Although our method is cross-validated with a leave-one-out approach (see Methods), we addressed the possibility that introducing more inputs to the decoder might lead to higher performances by chance. The first hint that this is not the case comes from the two Oddball datasets, where we did not observe such an increase (top panels in Fig. 5A). However, to provide further evidence that our method is not biased when using multiple inputs, we performed an additional control analysis. This analysis is based on the Face Perception dataset, as it showed the largest increase in performance with increasing number of channels. We shuffled the labels of the two experimental conditions, and recomputed the decoding performance with the same sets of channels used in this section (8 channel selection, and whole dataset). We repeated this procedure 24 times, and found that the mean decoding performance was consistent with chance level: 50.2% ± 0.2% (mean ± SEM) for the 8-channels set and 50% ± 0.2% for the 64-channels set. Therefore, the observed increase in performance cannot be attributed to any bias in the algorithm for high-dimensional data.

### 3.5 Spatiotemporal distribution of information

**Fig. 6:**
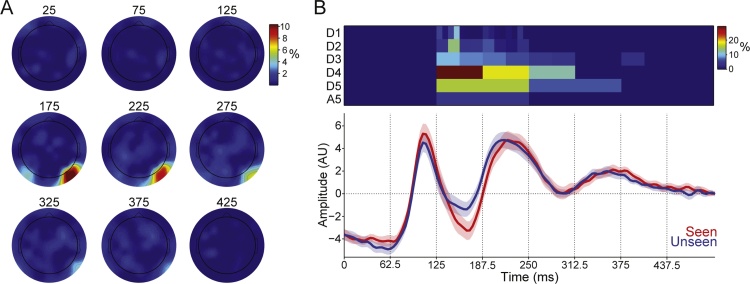
Tracking information fed to the decoders. (**A**) Average probability of selecting coefficients for classification in each of the 64 available channels at given post-stimulus times (indicated on the top of each head in ms). Plots show averages across all subjects. (**B**) Top panel displays the selection rate of each coefficient in electrode PO8. Each row comprises coefficients from a given decomposition level, as labelled. Coefficients were spanned in the horizontal axis along their corresponding time support in order to match time axis from ERPs traces. Bottom panel displays average ERPs for seen and unseen conditions across all subjects. Traces denote mean and shaded areas denote SEM.

As described in the previous section, considering multiple channels can add valuable information to distinguish experimental conditions from the ERPs. However, to get further insights about the brain processes involved in such conditions, we would like to know when and where this information comes from. Here, we show how this can be achieved on the face perception dataset, where we observed significant increases in decoding performance when considering larger sets of channels. In the multichannel implementation, selected wavelet coefficients may be associated with any channel, and will have a specific time support (i.e. the time spanned by the wavelet function). Because different coefficients might be selected for each trial in the leave-one-out protocol, we quantified the information provided by each coefficient by measuring the relative number of times that each coefficient was selected by our method. Then, we calculated the mean selection rate of the coefficients associated to each electrode at a given post-stimulus time (i.e., coefficients from that channel with time span including the given post-stimulus time). Fig. 6A shows the grand average (across subjects) results of this analysis. Note that for a given electrode, each post stimulus sample point has 6 associated wavelet coefficients (one for each decomposition scale); thus, each point in Fig. 6A refers to a selection rate averaged across 6 coefficients. As expected, coefficients from the PO8 electrode with a time span of ∼170 ms showed the highest selection rate. Complementing this information, in Fig. 6B (top panel) we show the average proportion of trials in which each individual coefficient from electrode PO8 was selected, along with the average of ERP traces for unseen and seen conditions across all subjects (bottom panel). Rows indicate decomposition levels (time scale) and the horizontal axis denotes time. Note that the most selected coefficients came from scale D4, especially the one with a time support between 125 ms and 187.5 ms, consistent with the N170 literature (Bentin et al., 1996; Rossion, 2014; Rousselet et al., 2007). Furthermore, this analysis shows that no information in the first 125 ms was ever used by the classifier, indicating that small differences in the ERPs during this time window (e.g., ∼100 ms positive peak) were not informative at a single-trial level.

## 4 Discussion

For several decades, ERPs have been routinely used both in clinical practice and in cognitive neuroscience. The standard approach is to elicit ERPs upon different conditions − different type of stimuli, different perceptual or cognitive responses, etc. − and then contrast responses to identify components that give a differential signal, thus being related to the process under study. Due to the low amplitude of the ERPs compared to the background EEG, these components are typically visualized after ensemble averaging and are then characterized in terms of their amplitude, latency and topography. Although this approach has provided major advances in our understanding of normal and pathological brain function (Freeman and Quian Quiroga, 2013; Niedermeyer and Silva, 2005), it is prone to miss, or even disregard a priori, relevant information. For example, the right occipitotemporal N170 response has been largely used to characterize brain processes related to face processing, as faces elicit a larger N170 compared to pictures of cars or other objects (Rossion, 2014). But could other ERP patterns, or combination of patterns, reflect face processing and contribute additional information to differentiate between these conditions? Could the activity in other electrodes, besides the ones traditionally explored based on a priori hypotheses, give valuable information?

For example, it might be possible to quantify differences in the shape of the ERPs by taking the area instead of the peak amplitude; to combine information of different peaks (e.g. taking peak-to-peak amplitudes); or to systematically explore all recording sites seeking for statistical differences across conditions. However, these approaches have two main caveats. First, they are ad-hoc and very time consuming, as the search and quantification of optimal patterns has to be done by hand on a case-by-case basis (and different optimizations will likely need to be used for different electrodes). Second, a systematic search for patterns and combination of patterns in different recording sites leads to statistical biases, as the obtained results need to be corrected for multiple comparisons.

In this study we proposed a new method, the WI method, to extract information from ERPs. The power of the method relies on the fact that: i) the wavelet transform provides a multi-resolution decomposition of ERPs, ii) the selection of wavelet coefficients produces a dimensionality reduction that captures the meaningful patterns, and iii) decoding provides a natural quantification of information. Compared to the standard analysis of peak amplitudes and latencies described above, WI has several advantages. First, it is completely unsupervised, so it does not require any tuning to capture information of specific patterns. Furthermore, the performance of the method does not depend on preprocessing steps, such as detrending and filtering. Second, patterns that are not necessarily reflected in features of single peaks or combinations of peaks are naturally captured by subsets of informative wavelet coefficients that are then fed into the decoding algorithm. In fact, the method has the potential to capture other temporal patterns, such as DC shifts, baseline crossings, etc. Third, the efficiency of the dimensionality reduction achieved by the selection of wavelet coefficients provides a natural way to combine information from multiple channels, without running into issues due to multiple comparisons, or computational problems that would be critical when analyzing high dimensional signals, what is also known as the ‘curse of dimensionality’ (Quian Quiroga and Panzeri, 2009). Fourth, the decoding approach gives a straightforward and objective quantification of information based on the single-trial ERP responses. Importantly, performance should not be evaluated only on the classification accuracy but also on its significance (Jamalabadi et al., 2016). Accordingly, in this current study we used permutation tests to assess statistical significance. Finally, the proposed method can be used to analyze any continuous neural recording, so besides scalp EEG and LFPs, it can be easily applied to intracranial EEG and MEG.

Alternative methods have applied principal components analysis (PCA) to capture the variance of both latency and morphology of single-trial ERP waveforms (Hu et al., 2011). However, PCA lacks temporal resolution and *a priori* information is required to define a time window for the estimation of the latency and amplitude of each ERP component on each single trial. In addition, PCA captures the direction of largest variability, which is not necessarily the same as the one of maximum separability (Quian Quiroga et al., 2004). Other methods linearly combine information from multiple sensors into a single channel that can be analyzed with conventional methods, such as temporal filtering, trial averaging, and frequency power analysis (Parra et al., 2005), but constraints need to be applied in order to select the weights in the linear combination. Moreover, by linearly combining multiple channels we would miss the opportunity of extracting different features from different channels.

The proposed method also presents some advantages when compared to other MVPA techniques. In particular, Schönauer et al. (2017) do not provide a feature selection step, although they propose to “condition” the data with pre-processing steps before applying a classification method. Other MVPA methods are applied across all electrodes for each time bin, providing a time course of decoding performance (Cauchoix et al., 2014, Cauchoix et al., 2016; Crouzet et al., 2015; King and Dehaene, 2014). However, this poses the problem of multiple comparison across individual time points that needs to be addressed, and prevents from combining information across different time bins. Our feature selection process based on the mutual information between each wavelet coefficient and the stimulus/condition allow us to reduce the dimensionality of all the data (time x frequency x electrodes) in a natural way.

In this work, we used a fixed number of wavelet coefficients to represent ERPs. However, one could employ a data-driven approach to define the set of coefficients to be used. For example, coefficients could be ranked by information and then iteratively added to the selected set until their joint information saturates. Importantly, in order to prevent biases this whole feature selection procedure must be based only on the training set. We tested this approach in our simulated dataset and found no differences in performance, but with a significant increase in the computational cost. Still, this approach has the potential to lead to better performances in some datasets with a large number of channels with redundant information. A MATLAB implementation of our method is available at https://www2.le.ac.uk/centres/csn/software, where the user can choose between both implementations.

We have shown with simulated data that WI gives information beyond the one provided by the amplitude and latency of evoked components. In this case, by construction, four different stimulus classes could be differentiated based on the shape (but not the amplitude) of the responses, something that was captured by the WI method. We also evaluated the performance of WI with four real datasets and showed that it gave significantly more information about the different conditions tested (target vs. non-target stimulus for the oddball datasets, and recognized vs. non-recognized for the other two datasets) compared to the one provided by the amplitude of the ERPs. The increase in performance when increasing the number of channels analyzed occurred in spite of the abovementioned fact that the increase in the dimensionality of the problem tends to diminish the ability to extract information from the data. In fact, there is typically a compromise when estimating information from real data: on the one hand, increasing the dimensionality of the data adds more information but, on the other hand, it impoverishes the ability to extract information (Quian Quiroga and Panzeri, 2009). The key feature of WI to avoid this problem is the dimensionality reduction achieved by selecting a set of informative coefficients.

Using a similar approach with spike train recordings we have previously shown that, contrary to other standard information estimation methodologies, the extracted information kept increasing when increasing the resolution used to bin the data, the length of the response considered, or the number of neurons (Lopes-dos-Santos et al., 2015). Furthermore, the dimensionality reduction achieved with a selection of informative wavelet coefficients provided significantly better results than other dimensionality reduction approaches, such as PCA or taking the time bins with largest information (without using wavelets) (Lopes-dos-Santos et al., 2015). The main difference between the current WI implementation and the one we previously proposed to extract information from spike trains is that the signal to noise ratio of the single trial ERP data is much lower compared to the one of spike trains and we therefore have to use sub-ensemble averages (instead of single trial traces) to estimate the information of the wavelet coefficients. More generally, the approach of selecting wavelet coefficients to extract information is reminiscent of a strategy used for spike sorting, namely, distinguishing spikes from different neurons based on their shapes (Quian Quiroga, 2012). In this case, the identity of the spikes is unknown a priori and it is therefore not possible to estimate information; however, a selection of wavelet coefficients having a multimodal distribution (i.e. reflecting information from more than one cluster of spikes) has shown to provide significantly better results than taking other ad-hoc spike features or PCA (Quian Quiroga et al., 2004).

Our method can easily deal with a large amount of data and number of channels. The fact that we can consider altogether the data in the whole response window, in all channels, in an unbiased way, and without multiple comparison issues, allows us to extract information beyond the one typically sought in hypothesis driven analyses, that focus on specific channels, time windows, and stereotypical responses. Moreover, the method can cope with subject by subject variabilities arising from variability in the precise location of the ERP sources or from misplacements in the recording sites. The ability to extract this information despite these variabilities is important for applications such as brain machine interface and neurofeedback. However, the proposed method is far from a black box approach, as it is possible to retrieve which specific information is associated with the obtained decoding performance. In particular, as shown in Fig. 6, the distribution of selected wavelet coefficients across subjects gives insights about the spatial location, scales (frequency bands), and time windows of the neural activity carrying relevant information to discriminate the different classes that are being contrasted in the experiment. In our dataset, our findings were consistent with the N170 potential associated to face perception, in terms of location, time, and frequency content.

### 4.1 Conclusions

In summary, we have presented a new unsupervised approach to analyze ERP recordings and extract (and localize) information that differentiates the conditions under study. This method not only extracts more information compared to other standard methods, but also opens possibilities of new paradigms and analyses that are not constrained, and potentially biased, by specific a priori hypotheses on how the evoked responses should look like, and where and when they should be searched for.
